# 15 million preterm births annually: what has changed this year?

**DOI:** 10.1186/1742-4755-9-28

**Published:** 2012-11-13

**Authors:** Mary V Kinney, Joy E Lawn, Christopher P Howson, José Belizan

**Affiliations:** 1 Saving Newborn Lives/Save the Children, Cape Town, South Africa; 2 Saving Newborn Lives/Save the Children, London, UK; 3 March of Dimes Foundation, White Plains, NY, USA; 4 Institute for Clinical Effectiveness and Health Policy (IECS), Dr Emilio Ravignani 2024, Buenos Aires C1414CPV, Argentina

## Abstract

Each year, more than 1 in 10 of the world’s babies are born preterm, resulting in 15 million babies born too soon. World Prematurity Day, November 17, is a global effort to raise awareness about prematurity. This past year, there has been increased awareness of the problem, through new data and evidence, global partnership and country champions. Actions to improve care would save hundreds of thousands of babies born too soon from death and disability. Accelerated prevention requires urgent research breakthroughs.

## 

Preterm birth has been the leading cause of neonatal mortality worldwide for about a decade. However, during 2012 new global estimates placed preterm birth as the number two cause of child mortality, behind pneumonia, with more than a million deaths each year [1]. Also during this year, the first national estimates of preterm birth prevalence for 184 countries found that 11% of the world’s babies are born preterm, before 37 completed weeks of gestation, resulting in 15 million babies born too soon [2].

The problem of preterm birth is shared by all countries. While 60% of preterm births occur in South Asia and sub-Saharan Africa [2], the United States and Brazil both rank among the top 10 countries with the highest number of preterm births. In the United States, for example, about one in 12 of all births is a preterm baby – nearly half a million each year. Yet, the burden remains highest in the regions with the fewest human resources. Of the 11 countries with preterm birth rates over 15 percent, all but two are in sub-Saharan Africa [2]. Thus, preterm birth is a global problem that requires collective and coordinated global action.

This week marks World Prematurity Day, November 17, a global effort to raise awareness about prematurity [3]. In celebrating, we pause to recognize all that has been achieved in the past year for preterm birth. To assess change over the last year, we have adapted the socio-political assessment framework of Shiffman and Smith, as applied to maternal health and newborn survival (Table 1) [4,5].

**Table 1:** **Changes for preterm birth since World Prematurity Day**, **17**^**th**^**November 2011**

**Issue characteristics**	
Data	***Cause of neonatal and child death*** estimates and trends, 2000–2010 (Global, regional and national) [1]
	- preterm birth now second leading cause of under-5 deaths, after pneumonia.
	- deaths from preterm birth complications reducing slower than other major causes of child death.
	***Preterm birth prevalence estimates*** for 184 countries for 2010 and trends 1990–2010 for 65 countries [2]
	- 14.9 million babies born preterm, > 60% in Africa and South Asia, highest rates in Africa.
	- Rates increasing over last 21 years in all but 3 of the 65 countries with trend estimates.
	***Preterm prevention analysis for 39 high income countries showed limited potential for prevention with current interventions ***[6]
	- Whilst rates appear to be recently leveling off in more than half of these 39 high-income countries, the largest national relative rate reductions so far are still under 5%
	Even with full coverage of 5 complex interventions, preterm birth rates can be reduced by only about 5%
Visibility	***Publications***
	*Born Too Soon*[7]
	*The Lancet* papers on preterm estimates, causes of death and prevention impact [1,2,6]
	***Media outreach***
	Major global media attention [8]
	Social media increased
	***World Prematurity Day***
	Four organizations spearheaded first World Prematurity Day in 2011
	More than 60 organizations participated in World Prematurity Day 2012, with events planned on over 40 countries.
**Ideas**	
Interventions	Global consensus of essential interventions for reproductive, maternal, newborn and child health including interventions for preterm birth. [9]
	Evidence reviews of preterm birth interventions [10]
	Inclusion of antenatal steroids on the United Nations Commission on Life-Saving Commodities [11]
Implementation	Cost effective, high-impact solutions becoming more widely recognized, with more priority given to countries scaling up, eg Kangaroo Mother Care and antenatal corticosteroids.
Research themes	Implementation research, especially for improved care of preterm babies.
	Discovery and development research, especially for prevention of preterm birth.
**Actor power**	
Partnership	More than 50 organizations joined to produce *Born Too Soon*.
	***Every Woman Every Child accountability*** system and 31 new commitments for preterm birth by global and local partners
Champions	Countries stepping forward as champions for preterm birth – eg Malawi, Uganda
	More global organisations paying attention to preterm birth
	Professional organisations showing leadership eg letter from FIGO president to all country members

Adapted from Shiffman J and Smith S [4].

In May 2012, more than 100 experts representing almost 50 agencies, universities, organizations and parent groups came together to produce *Born Too Soon*: *The Global Action Report on Preterm Birth* (Figure 1) [7]. This report presented the implications for the country estimates of preterm birth rates and identified evidence-based solutions for both prevention and care of the preterm newborn. As Secretary General Ban Ki-moon wrote in the Foreword of the report, the effort to reduce preterm births and deaths is an integral part of the *Global Strategy for Women*’*s and Children*’*s Health*. *Born Too Soon* generated widespread media attention reaching an audience of more than one billion people [8]. Since its publication, *Born Too Soon* has helped to catalyse a wider engagement of global partners and more vigorous and coordinated action in countries, linked to the accountability framework of the United Nations Secretary General’s *Every Woman Every Child* movement. With over 30 new commitments to Every Woman Every Child in the report made specifically on preterm birth, this topic has received one of the greatest numbers of commitments for a single theme. Even more importantly, it has stimulated action from global health agencies, governments, researchers, donor organizations and parent groups worldwide to address the problem of preterm birth.

**Figure 1:**
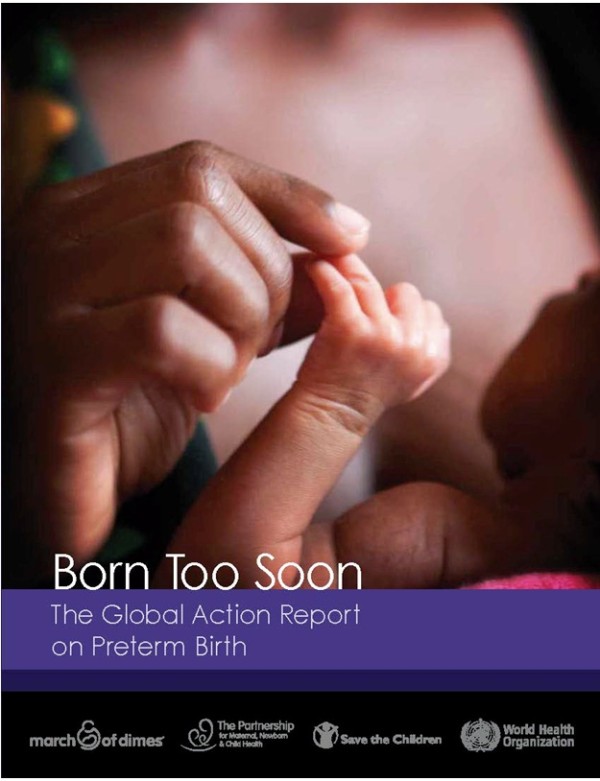
Born Too Soon: The Global Action Report on Preterm Birth.

World Prematurity Day was initiated in 2011 by the March Dimes and parent networks – The European Foundation for the Care of Newborn Infants (EFCNI), Africa-based Little Big Souls International Foundation and the National Premmie Foundation of Australia [3].

World Prematurity Day 2012 promises much greater participation from the growing movement of partners. Awareness activities, such as the Global Illumination Initiative--where cities are lighting landmarks and buildings in purple in honour of preterm babies and their parents--and the World Prematurity Day Facebook page, aim to increase knowledge of this issue, and inspire action. Parent groups have a particular power in promoting action and accountability.

Most importantly, multiple high burden countries are planning activities ranging from national events with government officials to local parent groups hanging posters at local hospitals. A number of countries will also announce major commitments to reduce preterm mortality. For example, Malawi, with the world’s highest estimated preterm birth rate, has a summit to champion change by continuing to scale up the use of kangaroo mother care and taking action to help ensure that antenatal steroids reach all who need them.

Evidence-based interventions for newborn survival have great potential to save newborn lives [12]. Improved care of preterm babies would result in immediate and substantial benefit [13]. More than 75 percent of the 1.1 million preterm babies who die each year could be saved without complex technological care. Two high priority interventions are Kangaroo Mother Care and antenatal corticosteroids that could save 450,000 and 400,000 premature babies each year, respectively, if made universally available [7]. As a result of the *Born Too Soon* report, coalitions of stakeholders dedicated to improving care for preterm babies have been established and efforts are underway to establish well-funded and collaborative research networks into the causes of preterm birth.

Prevention is the next frontier, especially for high-income countries where care is already being delivered to babies of extremely low gestational ages. An international team of experts has published a new analysis in *The Lancet* medical journal, which shows that even with high coverage of five interventions^a^, preterm birth rates in the 39 most developed countries could only be reduced by five percent by the year 2015 averting 58,000 preterm births and saving US$3 billion annually [6]. This major knowledge gap makes the case for a strategic and coordinated research effort to advance understanding of causes and mechanisms of preterm birth, leading to innovative solutions. Interventions cited in the *Born Too Soon* report, offer promise for prevention, particularly in lower-income countries where the causes of preterm birth may well be more amenable to prevention. These interventions, which can be delivered before and during pregnancy, include attention to birth spacing and addressing infections before and during pregnancy, such as malaria, HIV and syphilis [14,15].

The years ahead offer increased opportunities for more progress. As countries push towards the 2015 deadline for Millennium Development Goals 4 and 5 for child and maternal survival, and begin the dialogue for what comes next to address preventable deaths and promote sustainable development, progress in reducing preterm and newborn mortality have repeatedly been identified as major priorities [12].

Immediate attention should focus on low- and middle-income countries, which have the highest mortality burden, particularly among babies born between 32–37 weeks completed gestation and who would benefit most from simple and affordable care. Yet, we cannot forget the needs of affected babies and their families in higher-income countries, where survival is now at the thresholds of extreme gestational age and those who survive, often face a lifetime of significant disability [16].

The *Born Too Soon* report was written to advance epidemiology and clinical practice - yet was also intended for a general audience including policy makers and parents. Its contents will be reframed as a series of scientific papers next year in *Reproductive Health* during 2013. We hope that the forthcoming series in *Reproductive Health* will inform wider scientific and policy discussions and increase the pace of progress.

World Prematurity Day 2012 is more than one day of awareness – it is an acknowledgement of what has been achieved, especially in this last year, what still needs to be done and of the important message in *Born Too Soon* that we all have a role to play in addressing the problem of preterm birth. Academics are strengthening evidence, international organizations are supporting partners in countries, donors are allocating more funds although the baseline is very low [17], professional associations are improving care, governments are changing policies and practices and civil society and parent groups are mobilizing. Together we can and we are changing the future for every woman and every newborn, wherever they live or are born.

### Endnotes

^a^The five interventions included: smoking cessation, decreasing multiple embryo transfers during assisted reproductive technologies, cervical cerclage, progesterone supplementation, and reduction of non-medically indicated labour induction or caesarean delivery.
